# Active commuting to school: How far is too far?

**DOI:** 10.1186/1479-5868-5-1

**Published:** 2008-01-08

**Authors:** Norah M Nelson, Eimear Foley, Donal J O'Gorman, Niall M Moyna, Catherine B Woods

**Affiliations:** 1 Department of Sport, Culture and the Arts, University of Strathclyde, Glasgow, Scotland; 2 Institute of Technology Tralee, Co. Kerry, Ireland; 3 School of Health and Human Performance, Dublin City University, Dublin, Ireland

## Abstract

**Background:**

Walking and cycling to school provide a convenient opportunity to incorporate physical activity into an adolescent's daily routine. School proximity to residential homes has been identified as an important determinant of active commuting among children. The purpose of this study is to identify if distance is a barrier to active commuting among adolescents, and if there is a criterion distance above which adolescents choose not to walk or cycle.

**Methods:**

Data was collected in 2003–05 from a cross-sectional cohort of 15–17 yr old adolescents in 61 post primary schools in Ireland. Participants self-reported distance, mode of transport to school and barriers to active commuting. Trained researchers took physical measurements of height and weight. The relation between mode of transport, gender and population density was examined. Distance was entered into a bivariate logistic regression model to predict mode choice, controlling for gender, population density socio-economic status and school clusters.

**Results:**

Of the 4013 adolescents who participated (48.1% female, mean age 16.02 ± 0.661), one third walked or cycled to school. A higher proportion of males than females commuted actively (41.0 vs. 33.8%, χ^2 ^(1) = 22.21, p < 0.001, r = -0.074). Adolescents living in more densely populated areas had greater odds of active commuting than those in the most sparsely populated areas (χ^2 ^(df = 3) = 839.64, p < 0.001). In each density category, active commuters travelled shorter distances to school. After controlling for gender and population density, a 1-mile increase in distance decreased the odds of active commuting by 71% (χ^2 ^(df = 1) = 2591.86, p < 0.001). The majority of walkers lived within 1.5 miles and cyclists within 2.5 miles. Over 90% of adolescents who perceived distance as a barrier to active commuting lived further than 2.5 miles from school.

**Conclusion:**

Distance is an important perceived barrier to active commuting and a predictor of mode choice among adolescents. Distances within 2.5 miles are achievable for adolescent walkers and cyclists. Alternative strategies for increasing physical activity are required for individuals living outside of this criterion.

## Background

In recent years, there has been a dramatic worldwide increase in the prevalence of overweight and obesity among children and adolescents [1-5]. Health problems such as diabetes, metabolic syndrome and hypertension normally associated with adulthood are now being identified in adolescence [6]. There is an inverse relation between clustered cardiovascular [7] and metabolic syndrome risk factors [8] and physical activity among youth. Despite the well-established health benefits associated with regular physical activity, many young people do not meet recommended levels of physical activity. Currently, 65% of 15 – 17 year old Irish adolescents are not active for at least 60-minutes on four or more days per week. [9].

Walking and cycling to school provide a convenient opportunity to incorporate physical activity into the daily routine of children and adolescents. Children [10-13] and adolescents [14] who actively commute to school attain more minutes of daily physical activity than those who use motorized transport. Only 30% of Irish adolescents have reported that they actively commute to school [15].

Attempts to increase active travel and improve the walking environment for young people have resulted in a surge of resources and campaigns to develop safe walking and cycling routes to school [16,17]. School proximity to residential homes has been identified as an important determinant of active commuting among children [18]. More children walk or cycle to school as distance decreases [19-21]. Similar studies among adolescents are scarce [22].

Despite the fact that parents consistently cite distance as the number one barrier to their children actively commuting to school, [11,23,24] only 31% of US children, who live within 1 mile of their school choose to walk, and only 2% who live within 2 miles choose to cycle [23]. Among Irish adolescents, 22% of car users live within 1 mile, and 39% live within 2 miles of their school [9]. Where distance is not a barrier to active commuting, other factors such as convenient access to foot or cycle paths may inhibit walking or cycling.

Research focused only on individuals who live close enough to walk or cycle to school will increase our understanding of mode choices by removing distance as a confounding factor. The identification of a criterion distance within which children and adolescents walk or cycle to school will help promote active commuting, and encourage the appropriate inclusion of distance as a relevant determinant in research. The purpose of this study is to explore distance as a determinant of active commuting to school among adolescents. In particular, it seeks to identify if there is a criterion distance above which adolescents choose not to walk or cycle.

## Methods

All data were collected as part of the Take PART study (Physical Activity Research in Teenagers). Take PART was a cross-sectional study of participation levels, aerobic fitness, physical health indices, psychosocial and environmental determinants of physical activity in 15–17 year old Irish adolescents. Data were collected between February and May 2003–2005 using a one-stage cluster sampling procedure. Clusters were stratified based on school type (secondary, vocational & community colleges and community schools & comprehensives), gender and school location (urban, rural). A total of 82 schools were selected, and 61 agreed to participate. Subjects were eligible to participate if they were aged 15–17 yr, were not participating in state examinations, and obtained parental consent if under 16y, or provided their own consent if = 16 y. Eligible 15–17 year olds were recruited within each school and 50 participants were assessed during each 3-hour school visit, with a researcher participant ratio of 1:10. Standardized testing procedures were used throughout and extensive researcher training was undertaken to minimise potential sources of error in the physical measures and the administration of the questionnaire. Inter and intra-tester reliability for all measures was 0.7 or above.

The distance (miles) of the actual route travelled to school, and the usual mode of travel were assessed using a self-report questionnaire that was completed under supervision. Similar questions have previously been used in this age group [15]. Mode of travel responses were categorised as active commuting by foot or bicycle, or inactive commuting by car, bus or train. Adolescents who used mixed mode trips (for example walk/cycle to bus/train) responded based on the longest portion of their journey only. All adolescents who travelled by bus or train are assumed to undertake some walking or cycling to get to public transport but the amount of physical activity undertaken is unknown and this is a limitation of this study. Bus travel could have been public or private school buses. It is recognised that the mode of travel and determinants of trips to and from school may differ. To delimit this study, the return journey was not reported and this manuscript focuses on the journey to school.

A subset of participants (N = 272, mean age 15.93 ± 0.63 years, 51.6% male, 62.5% active commuters) self-reported distance travelled to school and drew their actual route on a detailed street level map (scale of 1:2500). The actual distance was measured using a map wheel (Scalex Corporation, California, U.S.A). Perceived and actual measurements were correlated (0.22, p < 0.001) and there was no significant difference between perceived and actual distance travelled (1.26 vs.1.23 miles, p = 0.774), indicating that perceived distance is a valid measurement tool. This was true for males (1.19 vs. 1.25 miles, p = 0.631) and females (1.32 vs. 1.22 miles, p = 0.356).

Barriers to active travel were assessed through an open response question. Individuals who travelled by car, bus or train were asked "Why do you choose not to walk or cycle?" Parental occupation was obtained to determine socio-economic status [25]. Participants were asked if they had a disability that restricted their participation in physical activity. Area of residence was classified as i) large city (>500,000 inhabitants), ii) suburbs or outskirts of a city (<500,000 but > 50,000), iii) town (<50,000) or iv) village (<5,000) [15].

### Data analysis

Data are presented as means, standard deviations and proportions where appropriate. The Pearson Chi square statistic was used to determine the relation between mode of transport and gender, and mode of transport and population density. Mann-Whitney tests were used to compare distance from school between males and females, active and inactive commuters, and between inactive commuters who cited distance as a barrier and those who did not. Differences in distance by population density were examined using a Kruskall-Wallis test and expected trends were examined using Jonckheere's test. Relevant effect sizes were calculated and reported as r-values. An r-value of 0.10, 0.30 and 0.50 represented small, medium, and large effect sizes respectively [26]. Distance was entered into a bivariate logistic regression model that predicted active versus inactive commuting to school, and controlled for gender, population density, socio-economic status and clustering at the school level. Open responses on barriers to active commuting were transcribed verbatim, categorised and themed using systematic content analysis [27-29]. Statistical analysis was undertaken in 2006 using SPSS for Windows, version 14.0.

## Results

### Descriptive characteristics of participants

In total, 4720 adolescents participated and 4013 completed all elements required for this study. Participants were excluded if they had a disability that affected their capacity to participate in physical activity (n = 344), or if they had incomplete data (missing responses for mode or distance, n = 398). A higher proportion of females had a disability (8.5 vs. 6.1% χ^2^(1) = 10.31, p < 0.001, r = -.047) and individuals with a disability had higher body mass index (22.94 vs. 22.5 kg^.^m^2^, t (368.88) = 3.58, p < 0.001, r = 0.18) than those who had no disability. There was no difference in mode of travel to school between respondents who had a disability and those who did not have a disability. The study design did not allow us to determine if disability influenced mode choice. A higher proportion of females were excluded due to incomplete data (58 vs. 42%, χ^2 ^(1) = 12.64, p < 0.0001, r = 0.05). There was no difference in age, socio-economic status or body mass index between respondents with a complete and those with an incomplete data set. All differences have small effect sizes and are unlikely to be substantive. Participant characteristics are presented in Table 1 (N = 4013 adolescents, mean age 16.02 ± 0.661, range 15–17 years).

**Table 1:** Participant characteristics

Characteristic	% (n)
Gender	
Male	51.9 (2083)
Female	48.1 (1930)

Age	
15	20.7 (829)
16	56.2 (2255)
17	23.1 (929)

Population density	
<5,000	6.1 (245)
<50,000	22.7 (910)
<500,000	29.9 (1199)
>500,000	41 (1646)

SES ^a^	
Non-manual	70.7 (2802)
Manual	29.3 (1211)

^a^SES = Socio-economic status. Non-manual includes professional, intermediate and junior non-manual occupations. Manual includes skilled, semi-skilled and unskilled manual occupations.

### Incidence of active commuting

Approximately one third of adolescents actively commute to school (Table 2). A higher proportion of males than females commute actively (41.0 vs. 33.8%, χ^2 ^(1) = 22.21, p < 0.001, r = -0.07) and more travel by bicycle (9.4 vs. 1%, χ^2 ^(4) = 156.86, p < 0.001, r = 0.19). The odds of active commuting to school are 36% greater for males compared to females (χ^2 ^(df = 1) = 22.26, p < 0.001).

**Table 2:** Mode of transport (% (n)) to school by gender

	Mode of transport		
	
	All	Male	Female
Walk	32.2 (1294)	31.7 (660)	32.8 (634)
Bike	5.3 (214)	9.4 (195)	1.0 (19)
Car	28.7 (1151)	26.3 (548)	31.2 (603)
Bus	33.1 (1329)	31.6 (658)	34.8 (671)
Train	0.6 (25)	1.1 (22)	0.2 (3)
All	100 (4013)	100 (2083)	100 (1930)

**Figure 1:**
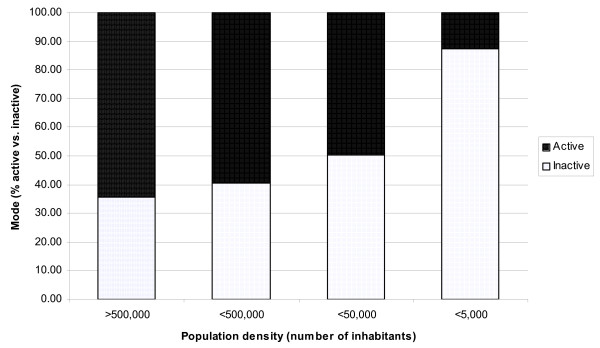
Decrease in proportion of active commuters as density decreases.

There is an inverse relation between population density and mode of travel to school (χ^2 ^(3) = 775.32, p < 0.001, r = 0.44). As population density decreases, the proportion of inactive commuters increases (Figure 1). Adolescents living in more densely populated areas have greater odds of active commuting than those in the most sparsely populated areas (χ^2^(df = 3) = 839.64, p < 0.001). Compared with village residents, the odds of active commuting are 12.6 (95% CI: 9.3–17.0), 10.1 (8.3–12.4) and 6.8 (5.7–8.2) times higher for those who live in cities, suburbs and towns respectively.

### Distance and mode choice

Table 3 displays the average distance travelled to school using each mode of transport. Adolescents who walk or cycle to school travel shorter distances (0.98 miles) than those who commute inactively (6.31 miles), (U = 292775.0, p < 0.001, r = -0.71). No gender differences were established in overall distance travelled to school. When analysed by mode, girls travel further by bicycle and boys travel further by train, however the number of females in sample size for these comparisons is very small.

**Table 3:** Average distance travelled (Mean ± St.dev) by gender

	Distance (miles)			
	
	All	Male	Female	Range
Walk	0.88 ± 0.75	0.89 ± 0.71	0.86 ± 0.79	0 – 5
Bike	1.62 ± 1.38	1.54 ± 1.33	2.46 ± 1.56 **	0.1 – 10
Car	4.46 ± 4.69	4.53 ± 4.43	4.40 ± 4.92	0 – 55
Bus	7.83 ± 5.69	7.85 ± 6.48	7.81 ± 4.79	0 – 75
Train	10.55 ± 8.59	11.57 ± 8.64	3.00 ± 2.00 *	0.75 – 30
All	4.31 ± 5.13	4.22 ± 5.33	4.40 ± 4.89	0 – 75

* p < 0.05. ** p < 0.01.

Distance travelled to school was influenced by area of residence (H(3) = 1043.69, p < 0.001). Jonckheere's test revealed a trend in the data: distance travelled to school increased as population density decreased (J = 3931634.5, z = 29.98, r = 0.47). In each density category, active commuters travelled shorter distances (Table 4).

**Table 4:** Average distance travelled by population density

Population density	Miles (Mean ± St.dev)			p ^a^
	All	Active	Inactive	

A big city (>500,000)	2.04 ± 3.85	1.02 ± 0.79	3.91 ± 5.97	<0.001
Suburbs (<500,000)	2.23 ± 2.99	1.02 ± 0.83	4.01 ± 3.98	<0.001
Town (<50,000)	3.01 ± 4.98	0.93 ± 0.88	5.08 ± 6.33	<0.001
Village/rural area (<5,000)	6.75 ± 5.33	1.04 ± 1.22	7.57 ± 5.20	<0.001

^a ^*p*-values for difference between mode types within each category. Bonferoni correction applied, significance level of .012 required

Over 80% of walkers live within 1.49 miles of their school. A further 7% live between 1.5 and 1.9 miles and 7% live between 2.0 and 2.49 miles of their school (Table 5). The proportions are similar for males and females, and in each population density category. Eighty four percent of cyclists live within 2.49 miles of their school. Similar proportions are evident among males and in each category of population density (data not presented). Females cycle longer distances to school than males (2.46 vs. 1.54 miles, U = 1074.5, p < 0.05, r = -.20). As a result less female cyclists live within 2.49 miles than males (57.9% vs. 86.7%).

**Table 5:** Distance travelled by mode of transport

Distance (miles)	Foot		Bicycle		Car		Bus		Train	
	
	% (n)	Cum %	% (n)	Cum %	% (n)	Cum %	% (n)	Cum %	% (n)	Cum %
0–0.49	25 (326)	25	7 (14)	7	2 (25)	2	0 (3)	0	0 (0)	0
0.5–0.9	28 (357)	53	16 (35)	23	4 (49)	6	1 (11)	1	4 (1)	4
1–1.49	**29 (378)**	**82**	28 (60)	51	14 (162)	20	3 (34)	4	4 (1)	8
1.5–1.9	7 (85)	89	13 (28)	64	6 (67)	26	2 (22)	5	0 (0)	8
2–2.49	7 (92)	96	**20 (43)**	**84**	**12 (143)**	**38**	**6 (74)**	**11**	**0 (0)**	**8**
2.5–2.9	1 (15)	97	3 (7)	87	4 (41)	42	1 (18)	12	0 (0)	8
3.0–3.49	2 (20)	98	6 (13)	94	12 (137)	54	7 (93)	19	8 (2)	16
3.5–3.9	1 (6)	99	1 (2)	94	2 (22)	56	2 (20)	21	0 (0)	16
4–4.49	1 (8)	100	2 (4)	96	7 (76)	63	7 (93)	28	12 (3)	28
4.5–4.9	0 (0)	100	4 (8)	96	0 (4)	63	1 (14)	29	0 (0)	28
>/= 5	1 (7)	100	0 (0)	100	37 (425)	100	71 (947)	100	72 (18)	100

Total	100(1294)		100 (214)		100 (1151)		100 (1329)		100 (25)	

Note. Cum % = cumulative percent. Bold, underlined = point of major change in proportions walking and cycling; car, bus and train marked for comparative purposes.

Approximately 4 in 10 car users and 1 in 10 bus users live within 2.49 miles of their school. A greater proportion of females (41%) than males (36%) take the car for journeys of ≤ 2.49 miles. In villages of <5,000 inhabitants, over 50% of car journeys and 80% of bus journeys to school are longer than 5 miles.

**Figure 2:**
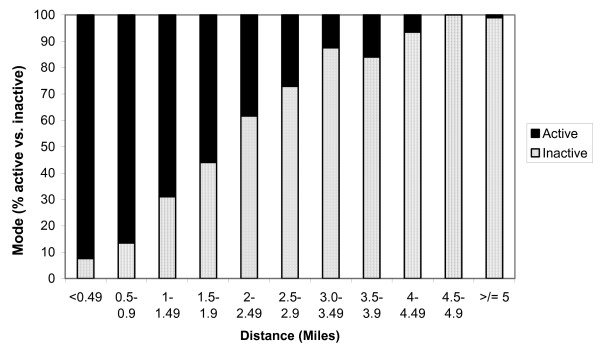
Decrease in proportion of active commuters as distance increases.

Distance predicts active commuting to school (χ^2 ^(df = 1) = 2591.86, p < 0.001), after controlling for gender, population density, socio-economic status and school clustering. A 1-mile increase in distance from school decreases the odds of active commuting by 71% (Table 6). The distance related shift from active to inactive mode is illustrated in Figure 2. Gender and density continue to influence the adjusted model. The odds of active commuting are 66% greater among males. Compared with village residents, the odds of active commuting are 2.1, 2.0 and 1.7 times higher for those who live in cities, suburbs and towns respectively.

**Table 6:** Logistic regression model

Variables Included	B (S.E)	Odds Ratio	95% C.I.	p
Constant	1.86 (1.21)	6.43		

Miles	-1.23 (.05)	.29	(.26, .32)	<0.001

Gender				
Male	.50 (.10)	1.66	(1.36, 2.01)	<0.001

Population density ^a^				
>500,000	.76 (.21)	2.13	(1.41, 3.23)	<0.001
<500,000	.69 (.15)	2.00	(1.49, 2.69)	<0.001
<50,000	.54 (.13)	1.71	(1.32, 2.23)	<0.001

Note. Adjusted for socio-economic status and clustering at school level. R^2^= 0.49 (Cox & Snell), 0.67 (Nagelkerke). 85.7% correctly predicted.

^a ^reference category is village, <5,000 inhabitants

### Perceived barriers to active commuting

Distance was the most commonly cited barrier to active commuting by males and females, in all categories of population density. Individuals who cited distance as a reason for inactive commuting lived significantly further from school (7.89 miles) than those who cited other reasons (2.86 miles), (U = 471671.5, p < 0.001, r = -0.56). Seventy four percent of adolescents who cited distance as a reason for inactive commuting lived ≥ 5 miles from school and 92.8% lived ≥ 2.5 miles from school.

Males and females in all categories of population density offered the same top four reasons for inactive commuting. After distance, time and intrinsic factors were the next most common reasons for inactive commuting (Table 7). Other factors hypothesised to influence mode choice, such as weather, heavy bags and safety, were reported less frequently than expected. Traffic related danger, unsafe environments and poor infrastructure for walking and cycling were cited by less than 5% of adolescents.

**Table 7:** Reasons for inactive commuting to school

Theme	% (n)	Categories
Distance	57.1 (1153)	Too far, too far to walk
Time	17.2 (347)	Would take too long, too early, would be late
Intrinsic factors	6.3 (128)	Laziness, inability to get up, couldn't be bothered, tiredness
Convenience	5.9 (120)	Parent passes school, lift offered, car is easier, parent works in school
Other	3.3 (62)	Mixed mode, walk home, not allowed, no bike, own car, bike broken
Weather	2.7 (54)	Too cold, weather, rain
Traffic related danger	1.7 (35)	Dangerous roads, busy roads, speeding traffic
Bags	1.7 (34)	Heavy bag, too many bags
Danger	0.5 (10)	Too dangerous, unsafe
Physical Environment	0.4 (9)	No paths, uphill

## Discussion

The incidence of active commuting to school amongst adolescents supports previous Irish research [15]. Internationally, rates vary considerably with higher incidence in European countries [11,30] compared to the United States [12,19,31], and among children [11,19] compared to adolescents [31]. Nonetheless, since the majority of Irish adolescents travel to school by bus or car they are missing out on important additional minutes [10-14] of potentially health-promoting physical activity. Based on differences in energy expenditure among active and inactive commuters, Tudor-Locke et al. (2003) estimate that young people who travel daily by sedentary means risk yearly weight gains of 2–3 lbs [32]. Research has yet to demonstrate that established physical health benefits of active commuting among adults [33-36] also apply to young people. One study to date has shown that cycling to school is associated with increased aerobic capacity compared with inactive travel modes [30].

Being female reduces the odds of active commuting by 36%. McMillan and colleagues (2006) reported a slightly higher value of 41.5% in 8–11 yr old girls indicating a reduced gender effect on mode choice among older youth [37]. Factors other than distance explain gender differences in mode; males and females travelled similar distances by foot, car and bus. Observed difference in distance travelled by bicycle and train are tentative due to small numbers of females using these modes. Many other factors might explain gender differences in mode, for example, perceptions of personal safety from real or perceived crime are predictors of recreational physical activity among adolescents, [38] especially females [39] and further research is required to identify if these factors also influence utilitarian activities such as active commuting to school. Though fewer females cycled to school, the distance they covered was further than males. This may reflect a high level of motivation among this minority. Research into the reasons for such low levels of cycling among female cyclists is required.

The further an adolescent lives from school, the less likely they are to walk or cycle. This extends previous findings in children [18-20] and signifies the importance of locating schools in or near residential communities. With the advancing sprawl around major cities in Ireland, and increasing evidence of the completion of new developments without the provision of schools and local amenities, such evidence is timely and should be considered in policy guidelines for urban planning and development. Among Irish adolescents the criterion distance for walking and cycling to school was ≤ 1.5 miles (2.4 km) and ≤ 2.5 miles (4.0 km) respectively. This indicates that 2.5 miles could be used as a general cut-off within which both walking and cycling to school are achievable. This criterion is greater than previously suggested adult guidelines [40] but lower than the 3.0-mile criterion required for government-subsidised transport to school for post-primary pupils in Ireland [41] and the U.K [42]. In Denmark, where rates of active commuting are 75%, 14–15 y old secondary school students must live a distance of ≥ 5 miles from school to avail of free transport [42].

The Healthy People 2010 initiative in the US seeks to increase the proportion of trips made by walking to school to 50% and by cycling to 5%, for children and adolescents living within one mile of their school [43]. This study provides evidence for the use of distance-related goals for promotion of active commuting, and reveals the need for population specific targets. Irish adolescents are already meeting U.S targets for 2010: approximately three quarters of Irish teenagers who live within one mile walk to school, and 8% within 2 miles cycle. The potential for modal shift in Ireland lies among the adolescents who live between 1.0 and 2.5 miles, and specifically in increasing the proportion who cycle to school. The 39% of car users, and 11% of bus users who live within 2.5 miles of their school are legitimate targets for change to active modes of travel. Among adolescents who reported distance as a barrier to active commuting, over 92% lived ≥ 2.5 miles from school and only 7% perceived 2.5 miles as too far to walk or cycle to school, indicating the acceptability of this criterion distance. Further research is required into the determinants of travel behaviours among adolescents who travel short distances by motorised means, and adolescents who perceive short distances as too far.

Not surprisingly we found that as population density decreases, the travel distance to school increases, resulting in fewer adolescents actively commuting. Since fewer adolescents in areas of low density live within the proposed 2.5-mile criterion, this reduces the likelihood of active commuting making a contribution to daily minutes of physical activity, except among the highly motivated. Health promotion initiatives for low-density areas should focus on alternative strategies for increasing physical activity. In areas where transit supply is adequate, previously suggested promotion efforts could be applied to target these individuals including mixed mode travel [44] and "walk a stop" campaigns [45]. Mixed mode trips and using different modes for journeys to and from school were not examined in this study, but should be examined in future research as those who travel by bus or train may undertake more physical activity than those who walk or cycle the full distance to school.

Self reported barriers to active commuting were explored in this study. Similar to research among children, [11,23,24] distance was established as the most important perceived barrier among adolescents. In addition, new previously unconsidered reasons emerged. Lack of time, intrinsic factors such as laziness and tiredness, and convenience were more important than weather, traffic related danger or heavy bags. Two potential reasons for the difference from previous research are considered. Firstly, this study measured only the journey to school, for which issues like time and convenience may be considerably more important than they are for the return journey. Similarly, issues surrounding tiredness and laziness may be related to the adolescent's motivation to go to school, and these are unlikely to affect the return trip. Secondly, previous research in this area was conducted with younger children, and was based on parental report of barriers. It is hardly surprising then that traffic related danger or heavy bags were more commonly cited. This research suggests that the determinants of active travel differ from childhood to adolescence and highlights the need for adolescent-specific research. Adolescents who cited distance as a barrier lived further from school than those who gave other reasons for inactive commuting. Objective measurements of distance travelled are required to identify if distance is a real or a perceived barrier to active travel.

The current analysis is based on self-reported distance. Previous research among adults has shown a tendency to over-estimate distance [46] however there was no difference between self-reported and actual distance among adolescents in this sample, increasing confidence in the chosen criterion. In addition, perceived distance accounted for 49–67% of the variance in commuting behaviour suggesting that it is an important and relevant variable, possibly regardless of actual distance. Inaccurate perceptions of distance may themselves influence mode choice. One third of parents who perceived distance as a barrier to their children's active commuting, actually lived within 0.8 km of the child's school [19]. This finding illustrates the importance of perceptions as a determinant of behaviour. As long as it is unknown whether perceptions or actual measurements are more important, [47] both should be considered. Research is required comparing perceived to actual distance, and actual distance as a predictor of mode choice. In addition, research examining how to reduce inaccurate perceptions of distance is required to fully overcome distance as a barrier to active travel.

## Conclusion

To our knowledge this is the first study to assess distance as a determinant of active travel to school among adolescent boys and girls. Distance emerged as the most important perceived barrier to active commuting, and a predictor of mode choice. Future research considering the determinants of active travel among adolescents should apply a 2.5-mile criterion within which active commuting to school is achievable. This will improve the ability to explain mode choice by removing distance as a confounding factor and thus advance our understanding of this important physical activity behaviour. Active commuting interventions should target individuals who live within 2.5 miles of their school. Promotion efforts for teenagers who live ≥ 2.5 miles from their school should emphasise alternative strategies to increase physical activity. When planning new communities, schools should be located within 2.5 miles of residential areas.

## Competing interests

The author(s) declare that they have no competing interests.

## Authors' contributions

CW, NM and DO'G conceived and designed the study. NN and EF coordinated data collection. NN carried out data analysis, interpretation and drafted the first outline of the paper with assistance from CW. All authors revised the manuscript and approved the final version. The study was conducted while NN and EF were at Dublin City University.
